# Preoperative Intra-abdominal Sepsis, Not Penetrating Behavior Itself, Is Associated With Worse Postoperative Outcome After Bowel Resection for Crohn Disease: A Retrospective Cohort Study

**DOI:** 10.1097/MD.0000000000001987

**Published:** 2015-11-13

**Authors:** Tenghui Zhang, Jianbo Yang, Chao Ding, Yi Li, Lili Gu, Yao Wei, Lei Cao, Jianfeng Gong, Weiming Zhu, Ning Li, Jieshou Li

**Affiliations:** From the Department of General Surgery, Jinling Hospital Affiliated to Southern Medical University (TZ, JG); and Department of General Surgery, Jinling Hospital, Medical School of Nanjing University, Nanjing, Jiangsu, China (JY, CD, YL, LG, YW, JG, WZ, NL, JL).

## Abstract

It is generally believed that penetrating behavior is associated with worse surgical outcomes in Crohn disease (CD). We hypothesized that intra-abdominal sepsis (IAS), but not penetrating behavior itself, contributes to postoperative morbidity in patients undergoing bowel resection for CD.

Patients who underwent surgery from April 2010 to April 2014 were retrospectively identified from a prospectively maintained database. Demographic information and preoperative and operative data were collected. The outcomes following surgery in patients who had penetrating disease with or without IAS versus nonpenetrating CD were compared.

Of 288 patients, 180 had penetrating CD, including 54 who had IAS. Preoperative characteristics were similar between the groups, except for serum albumin, abdominal drainage, and prior bowel resection. Patients with penetrating CD with IAS were more likely to have a stoma, surgical site complications, postoperative IAS complications, and major complications than patients with penetrating CD without IAS or nonpenetrating CD. There were no significant differences between patients with penetrating CD without IAS and nonpenetrating CD. The postoperative outcome was strengthened after propensity-score matching analysis. Moreover, penetrating CD with IAS (odds ratio [OR], 13.034; *P* = 0.004) is a risk predictor for major postoperative complications, and preoperative serum albumin (OR, 0.095; *P* = 0.002) and preoperative enteral nutrition (OR, 0.203, *P* = 0.049) are protective.

Penetrating CD without IAS did not adversely affect postoperative outcome after bowel resection compared with penetrating CD with IAS. These results may revise the notion that all patients with penetrating CD have worse postoperative complications.

## INTRODUCTION

Despite improvement of medical care and introduction of anti-tumor necrosis factor (TNF) therapy, a substantial number of Crohn disease (CD) patients require bowel resection. According to the Montreal classification, CD can present as penetrating (B3) or nonpenetrating (B1 and B2). The present phenotypic classification of CD plays an important role in patient management and may help to predict the clinical course.^1^ The influence of disease behavior on long-term course of CD after surgery has been investigated, and penetrating CD seems to be associated with a higher recurrence rate compared with nonpenetrating lesions.^2^ The impact of disease behavior on short-term postoperative outcome is an area of concern. It was generally believed that patients undergoing surgery for penetrating CD experienced a high rate of postoperative complications. In a series of 550 patients that had undergone 633 operations for primary or recurrent CD, Kanazawa et al^3^ reported that penetrating disease significantly increased the risk of postoperative intra-abdominal septic complications. Bellolio et al^4^ reported that 293 patients with penetrating CD were more likely to require a more complex procedure, higher ileostomy rate, and longer postoperative stay.

Penetrating CD can present with intra-abdominal abscesses, phlegmon, free perforations with generalized peritonitis, or fistulas only. Therefore, some cases of penetrating CD manifest only as enterocutaneous or internal fistula with no obvious intra-abdominal sepsis (IAS). In addition, with the advancement of imaging and interventional techniques, intra-abdominal abscess and phlegmon due to penetrating CD can be detected early and controlled prior to surgery. Zerbib et al^5^ found that preoperative management for penetrating CD allowed for ileocecal resection with low rates of postoperative morbidity and fecal diversion.

We hypothesized that the increased postoperative morbidity in penetrating CD is not due to the disease behavior itself, but rather is a consequence of IAS. The objective of this retrospective review was to compare the intraoperative and postoperative outcomes of patients who had penetrating CD with or without IAS versus nonpenetrating disease.

## MATERIALS AND METHODS

### Demographic Data

All patients who underwent bowel resection for CD at the Department of General Surgery in Jinling Hospital from April 2010 to April 2014 were identified from a prospectively maintained database. Patients who underwent operations for abscess drainage without bowel resection, closing of ileostomy or colostomy, bypass surgery, reoperation for postoperative complications, and operations for reasons other than CD were excluded from analysis. Patients with an abscess occurring beyond 1 month after surgery were also excluded, as were those who underwent surgery for rectovaginal fistula or isolated perianal CD lesions. The study was approved by the Ethics Committee of Jinling hospital.

Demographic data such as body mass index, smoking habits, preoperative information and medical therapy, Montreal classification, and intraoperative and postoperative findings were collected from the database. Missing or incomplete data were collected by reviewing hospital medical records or by contacting patients directly, and noted accordingly in the results when not available.

### Classification of Disease and Definitions

Crohn disease was classified as penetrating or nonpenetrating based on the preoperative, intraoperative, and pathological findings. Patients who had an abscess, phlegmon, free perforation, or fistula diagnosed preoperatively, at the time of surgery, or pathologically were considered to have penetrating disease (B3). All other patients were considered to have nonpenetrating disease (B1 or B2). If stricturing and penetrating complications were discovered at the same time, the case was classified as penetrating.

Intra-abdominal abscesses were all detected by computed tomography (CT) and defined as an extraluminal fluid collection that was documented by CT, needle aspiration of pus, and/or operative finding of a pus-filled extraluminal cavity. Phlegmon was identified according to the radiological description of an inflammatory mass adjacent to an area of inflamed bowel. Stricturing phenotype was defined as stenosis by imaging or endoscopy, with associated prestenotic dilation or clinical symptoms consistent with partial bowel obstruction.

Patients were categorized into 1 of the 3 groups. Nonpenetrating CD included stricture or obstruction caused by fibrosis or failure of medical therapy, whereas penetrating CD without IAS included enterocutaneous or internal fistula, or resolved abscess and/or phlegmon. Penetrating CD with IAS was defined as unresolved abscess, phlegmon, or generalized peritonitis. Abscess and phlegmon with treatment failure were also categorized in this group.

### Preoperative Management and Surgical Techniques

Enteral nutrition (EN) using a preferentially elemental diet was applied before surgery in patients with nutrition risk (NRS 2002 ≥ 3) or intestinal stricture. Patients with complete intestinal obstruction or severe colitis received total parenteral nutrition (TPN) before surgery. For patients with IAS, TPN was given and then changed to EN after IAS was controlled.

Patients with symptoms and signs of IAS received intravenous antibiotic therapy and adapted to bacteriological results. Steroids and anti-TNF therapy were withdrawn if possible before surgical resection. Immunosuppressants and 5-aminosalicylic acid were stopped on hospitalization.

Abdominal imaging (CT enterography or magnetic resonance enterography) was routinely performed prior to surgery. For free perforation, an emergency operation was performed. For abscess, percutaneous drainage (PD) with ultrasound or CT guidance was attempted before surgery if possible. Phlegmon was treated before surgery with bowel rest, TPN, and intravenous antibiotics.

Phlegmon and/or abscess resolution was defined as confirmed radiographic resolution, or alternatively, if no radiographic imaging were available after treatment, complete resolution of the patient's symptoms, allowing for elective surgery and negative findings intraoperatively.

Treatment failure was categorized as technical failure or clinical failure, as defined by Bafford et al^6^ (1) technical failures—those in which drainage was attempted, but not completed because of failure to aspirate fluid from abscess collections; and (2) clinical failures—those with persistent signs and symptoms of sepsis and incomplete abscess resolution on imaging, necessitating urgent/semiurgent surgery.

All operations were undertaken by a stable team of surgeons. In both open and laparoscopic procedures, a functional end-to-end or side-to-side stapled anastomosis or stoma was selected by the surgeons. For colorectal anastomosis, a stapled side-to-end or end-to-end anastomosis was performed. In cases of internal fistula, the diseased and victim organ (bowel or bladder) were divided. The defect of the victim organ was then closed using 4–0 Vicryl absorbable sutures. A stoma was constructed if there was a residual abscess, the dissection was difficult, or the patient's nutritional status was poor.

### Outcome Measurement

Both intraoperative and postoperative outcomes were assessed including duration of surgery, need for a temporary ileostomy or colostomy, postoperative complications (Clavien–Dindo classification),^7^ estimated blood loss at surgery, need for reoperation, and duration of postoperative hospital stay. All complications were defined as those occurring within 30 days after surgery or before discharge, whichever time frame was longer. Postoperative IAS included abdominopelvic abscess, peritonitis, or anastomotic leak, whereas surgical site complications included IAS, wound dehiscence, local fistula, or wound infection. Major complications were defined as Clavien–Dindo Grade III–V.

### Statistical Analysis

Variables were assessed for normality using the Levene test. Student's *t* test was used for normally distributed values which are presented as mean and standard deviation, and χ^2^ or Fisher's exact test was used to compare the categorical data, as appropriate. One-way analysis of variance (ANOVA) was used for multiple group comparisons. A propensity score analysis as a superior and more refined statistical method was performed to adjust for potential baseline confounding variables between groups. The “PSMatching” and the “REssentials for SPSS” packages were used to perform a propensity score matching analysis. Multivariate analysis was performed to identify the independent risk factors for postoperative major complications and reported as odds ratios (OR) with 95% confidence intervals (CI). For all analyses, *P* < 0.05 was considered statistically significant. SPSS version 20.0 software (IBM, Armonk, NY) was used in all analyses.

## RESULTS

A total of 288 patients (27.8% women) were included: 180 (54 with IAS) with penetrating and 108 with nonpenetrating CD. Demographic data are shown in Table 1. Major characteristics did not differ significantly between the groups except for preoperative serum albumin (*p*_1_ = 0.019, *p*_2_ = 0.037, *p*_3_ < 0.001) and prior bowel resections (*p*_1_ < 0.05).

**TABLE 1:**
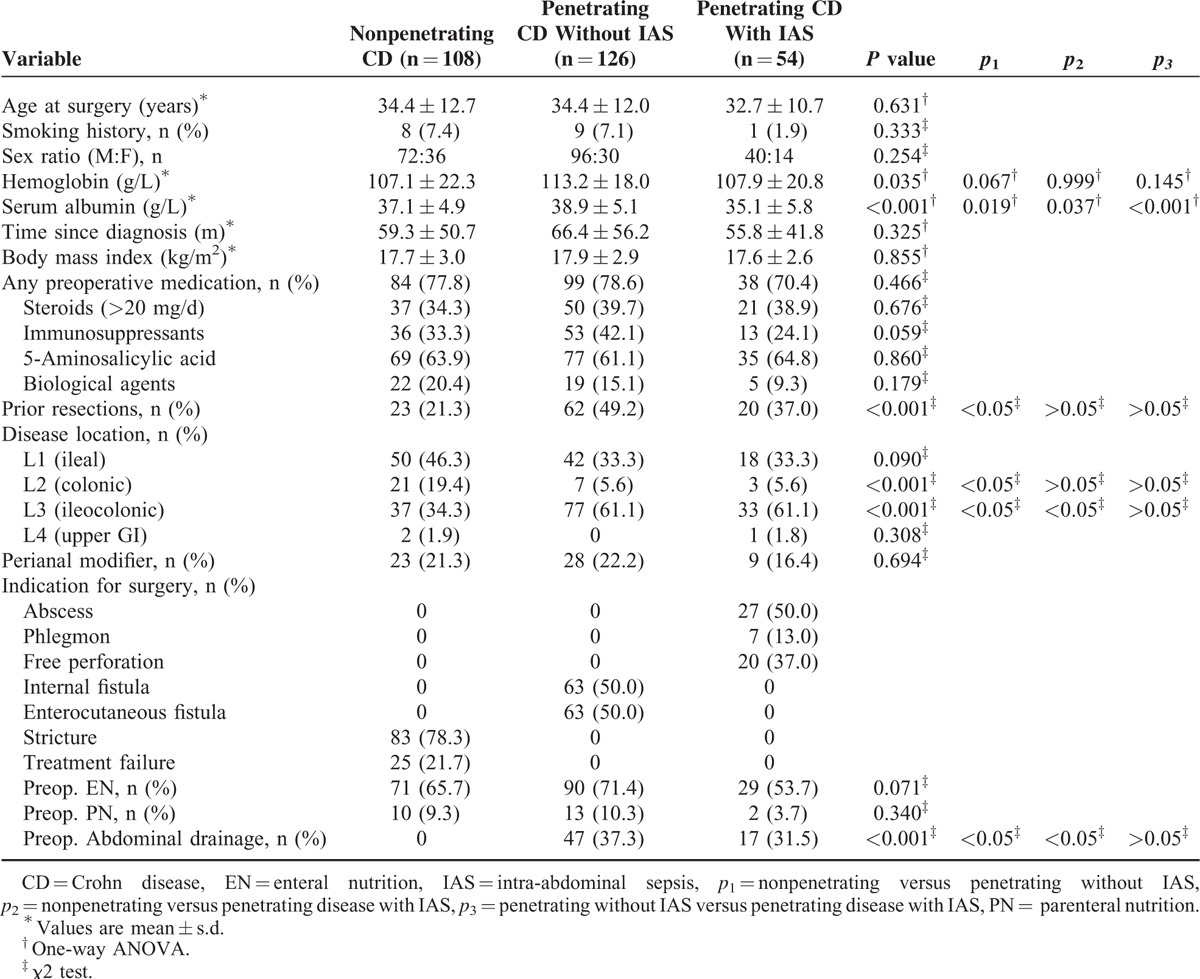
Preoperative Characteristics and Management

Patients with penetrating CD with IAS were more likely to have open surgery (*p*_*1*_*, p*_*2*_, *p*_*3*_ < 0.05). The surgical procedures are listed on Table 2. The duration of surgery and postoperative stay did not differ among the groups. Patients with penetrating CD with IAS were more likely to require a temporary stoma than those with penetrating CD without IAS and nonpenetrating CD (*p*_*2*_, *p*_*3*_ < 0.05), but the stoma rate in patients with penetrating CD without IAS was comparable to that in patients with nonpenetrating CD (*p*_1_>0.05). There was more blood loss (92.5 vs 132.9 mL, *p*_1_ < 0.001, 92.5 vs 132.9 mL, *p*_*2*_ *=* 0.002) in patients with penetrating CD with or without IAS than in patients with nonpenetrating CD during surgery.

**TABLE 2:**
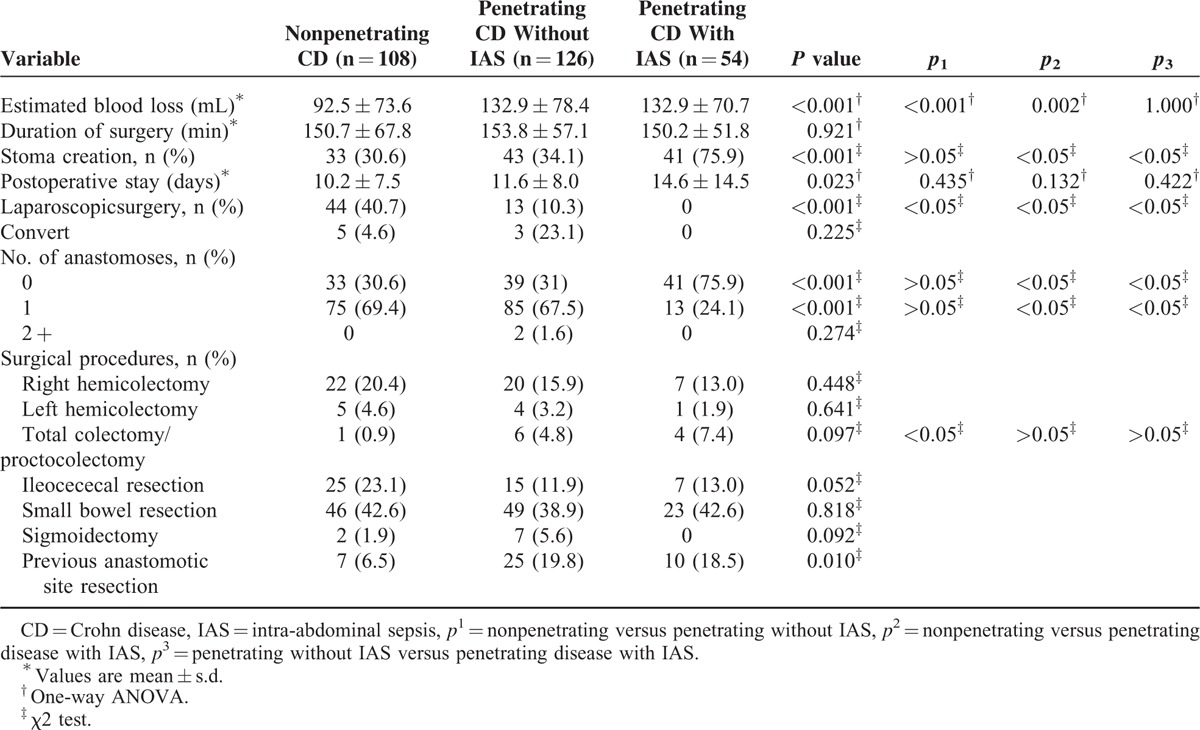
Operative Data

Patients with penetrating CD with IAS were more likely to have more overall postoperative complications (*p*_2_ < 0.05) than the other 2 groups, though there was no significant difference between patients with penetrating CD with IAS and without IAS (*p*_*3*_>0.05) (Table 3). Major complications (*p*_2_, *p*_*3*_ < 0.05), postoperative IAS complications (*p*_2_, *p*_*3*_ < 0.05), and surgical site complications (*p*_2_, *p*_*3*_ < 0.05) differed between patients with penetrating CD with IAS versus patients with penetrating CD without IAS and patients with nonpenetrating CD. Uneventful operative outcomes were significantly lower (*p*_2_ < 0.05) in patients with penetrating CD with IAS than patients with nonpenetrating CD. Nine patients with penetrating CD with IAS had postoperative abscess or leak, compared with 3 patients with penetrating CD without IAS and 2 patients with nonpenetrating CD. Endoscopic hemostasis was performed in 1 patient with penetrating CD without IAS. Two patients with penetrating CD needed reoperation, owing to anastomotic leak (1 patient without IAS) and gastrointestinal bleeding (1 patient with IAS). One patient with anastomotic leak and 1 with stoma stricture in the nonpenetrating CD group needed reoperation. Two patients with penetrating CD with IAS required intensive care for renal insufficiency and methicillin-resistant *Staphylococcus aureus* enteritis. No patients died in any of the groups.

**TABLE 3:**
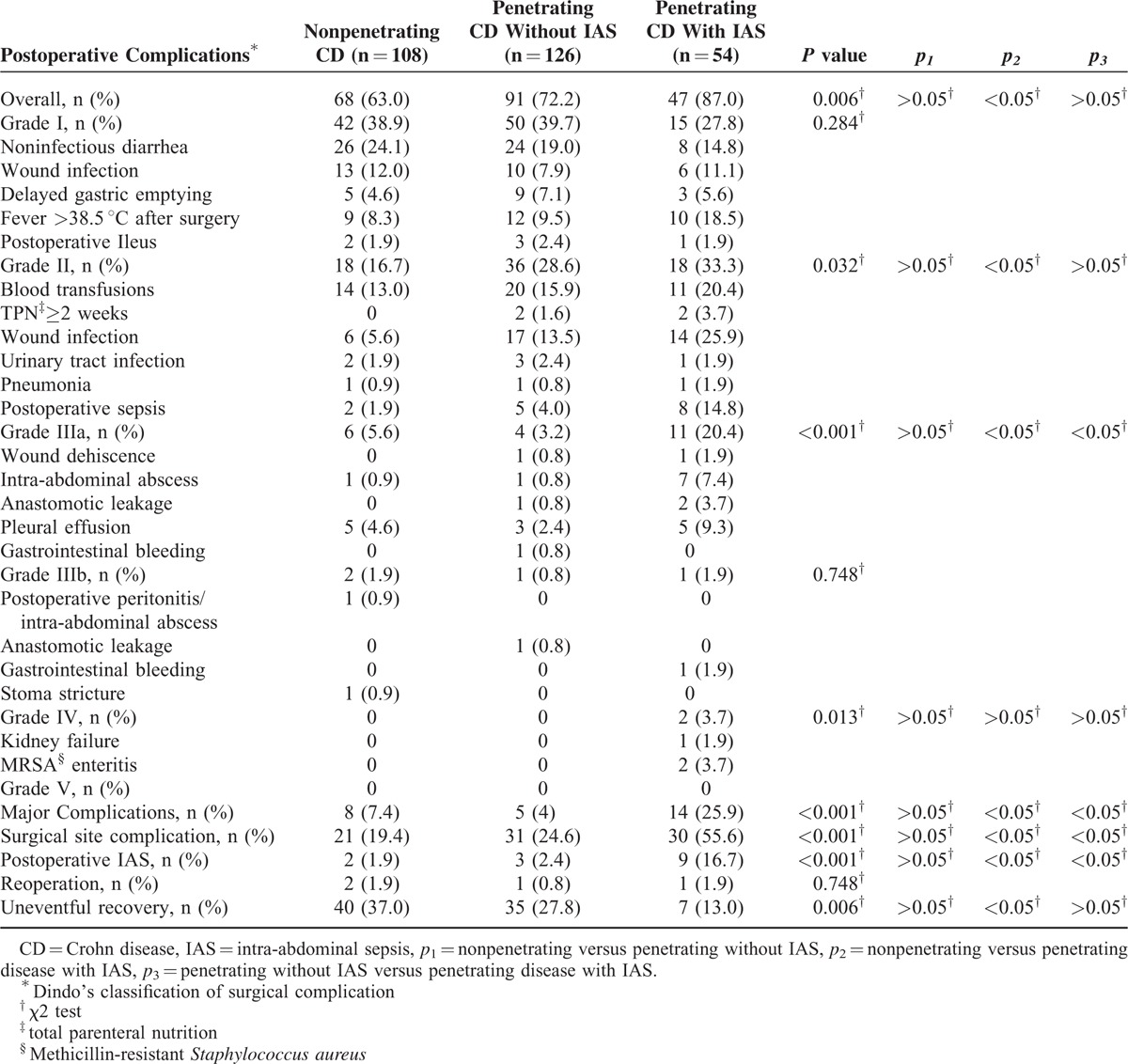
Postoperative Outcomes

After propensity-score matching, preoperative serum albumin (38.3 vs 39.3 g/L, *P* = 0.118) and prior bowel resection (*P* = 0.215) were not significantly different between nonpenetrating CD (n = 70) and penetrating CD without IAS (n = 70). There were no significant difference in major postoperative complications (*P* = 1.000), postoperative hospital stay (*P* = 0.147), surgical site complication (*P* = 0.111), postoperative IAS complications (*P* = 1.000), and proportion of stoma (*P* = 0.353), even if more estimate blood loss (*P* = 0.009) in penetrating CD without IAS compared to nonpenetrating CD (Table 4). Furthermore, after adjustment for preoperative parameters in penetrating CD with IAS versus nonpenetrating CD (n = 48 and n = 48, respectively) and penetrating CD with IAS versus nonpenetrating CD (n = 43 and n = 43, respectively), patients with penetrating CD with IAS were still more likely to have a stoma (*P* < 0.001), longer postoperative hospital stay (*P* = 0.038), surgical site complications (*P* < 0.001), postoperative IAS complications (*P* = 0.036), and major complications (*P* = 0.016) than nonpenetrating CD (Table 5) and more likely to have a stoma (*P* < 0.001), surgical site complications (*P* < 0.001), and major complications (*P* = 0.007) than patients with penetrating CD without IAS (Table 6).

**TABLE 4:**
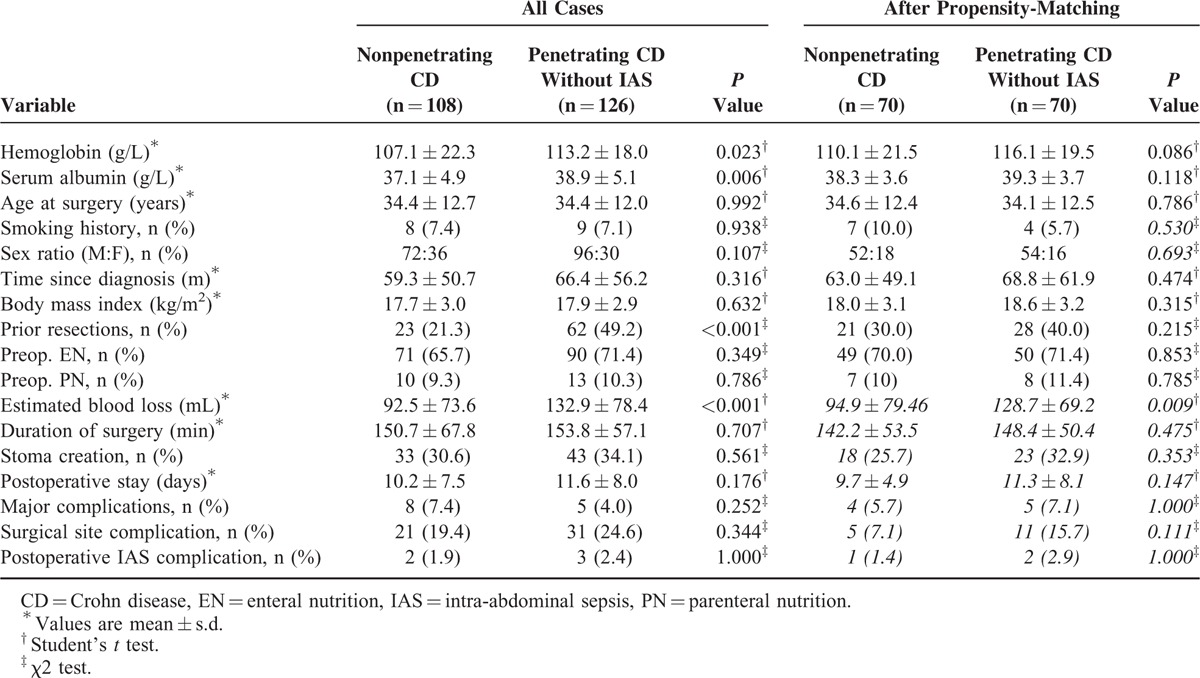
Preoperative and Postoperative Data in Patients With Penetrating CD Without IAS and Nonpenetrating CD

**TABLE 5:**
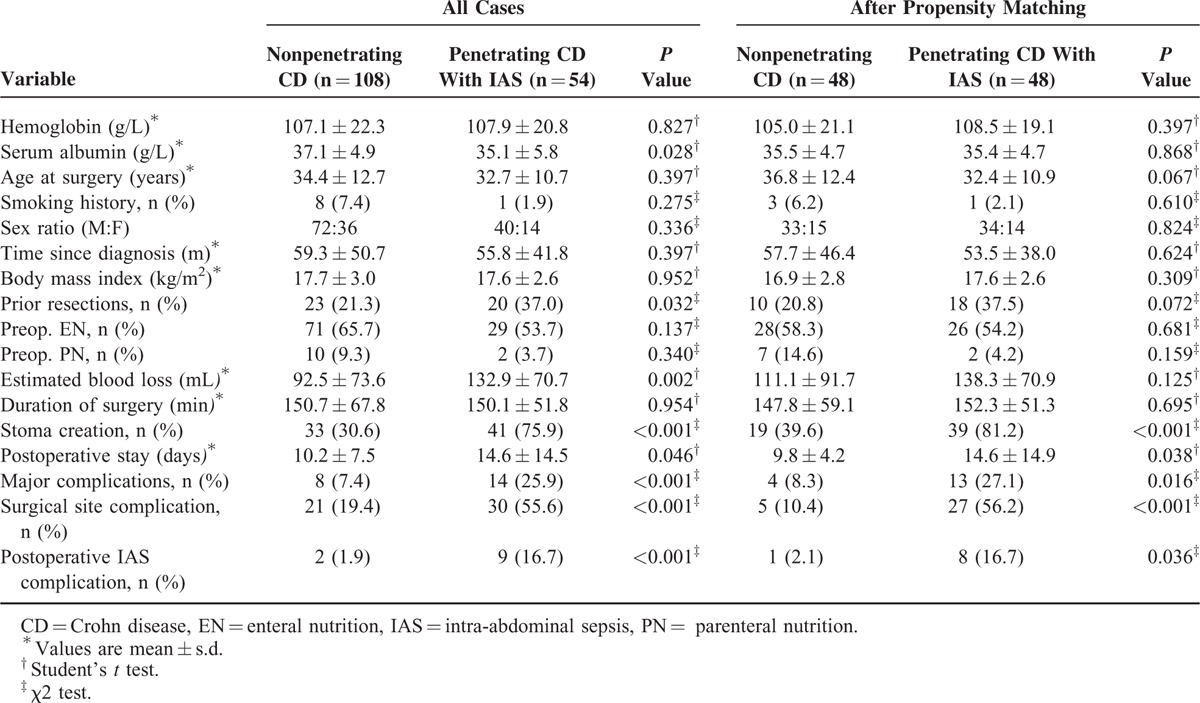
Preoperative and Postoperative Data in Patients With Penetrating CD with IAS and Nonpenetrating CD

**TABLE 6:**
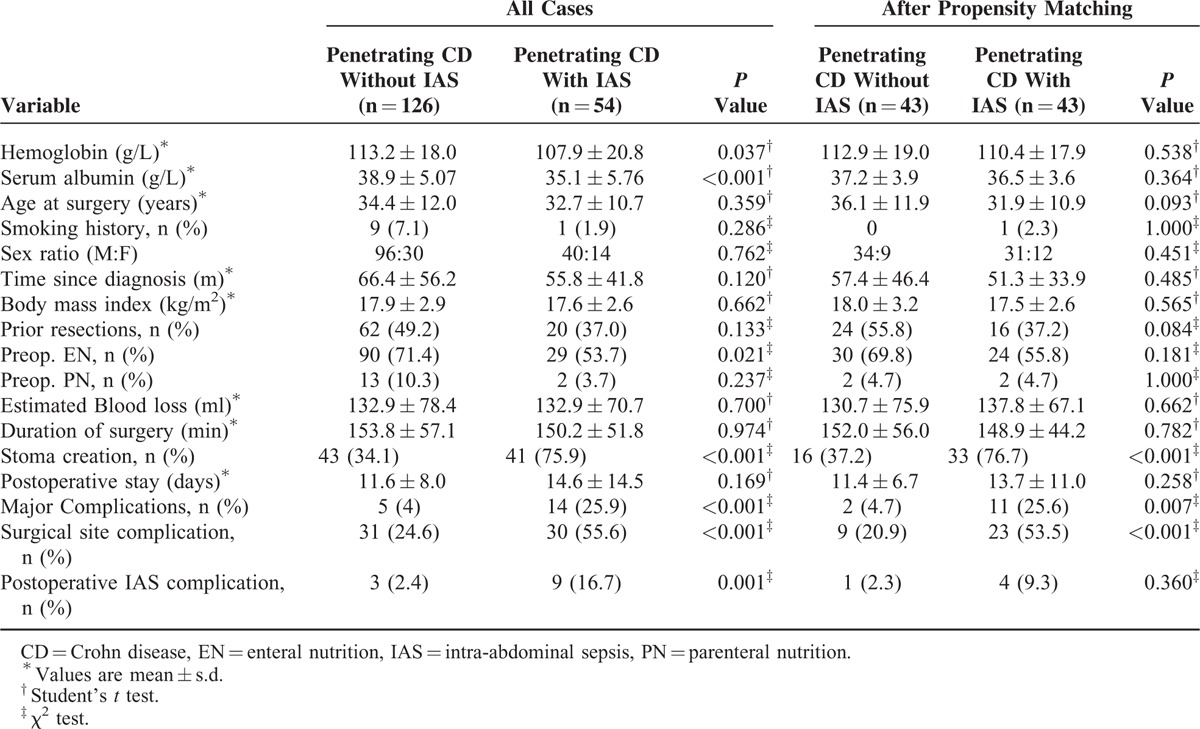
Preoperative and Postoperative Data in Patients With Penetrating CD With and Without IAS

Multivariate analysis showed that postoperative major complications were significantly associated with preoperative serum albumin (>35 g/L) (OR, 0.095; CI, 0.02–0.43), preoperative EN (OR, 0.203; 95% CI, 0.04–0.99), and penetrating CD with IAS (OR, 13.034; 95% CI, 2.22–76.52) (Table 7).

**TABLE 7:**
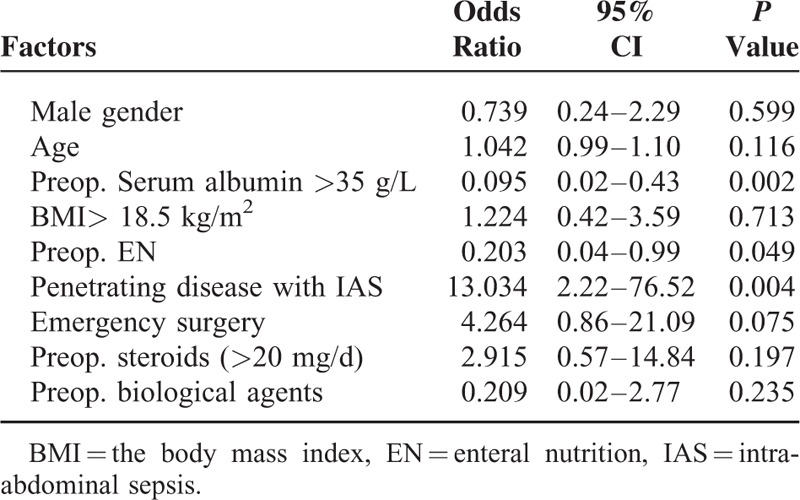
Multivariate Analysis of Risk Factors Associated With Major Complications After Surgery for Crohn's Disease

## DISCUSSION

The present study revealed that penetrating CD is not predictive of adverse postoperative outcome in patients requiring bowel resection. Indeed, the sequela of penetration, IAS, is a major risk factor of postoperative complications in penetrating CD. Although this was a retrospective study, patients were identified from a prospectively maintained database. The study included only patients undergoing bowel resection by a stable team of surgeons. The definition of penetrating and nonpenetrating CD was based on the Montreal classification, which defines current A (age at diagnosis), L (location), and B (behavior) status. A relatively high proportion of patients (62.5%) in the current cohort were classified with penetrating CD, possibly owing to the fact that this institution is a referral center for inflammatory bowel disease in China.

Overall preoperative characteristics for nonpenetrating compared with penetrating CD were similar, except for albumin and prior bowel resection. Patients with penetrating CD without IAS had a better preoperative nutritional status than those with nonpenetrating CD and penetrating CD without IAS. This was possibly because successful control of IAS delays surgery and allows for a longer duration of preoperative nutrition support, whereas patients with nonpenetrating CD had a poor nutritional status owing to obstruction caused by fibrosis or failure of symptoms to respond to medical therapy. In addition, patients with penetrating CD with IAS often need emergency or semiemergency surgery, leaving not much time for preoperative nutritional therapy.

Previous studies have shown that penetrating CD is associated with a worse outcome compared with nonpenetrating disease. In a retrospective review of 434 patients with perforating ileocolic resection for CD, Bellolio et al^4^ found that patients with perforating CD were more likely to require a more complex procedure, postoperative abscess or leak, and such as ileostomy, to postoperative abscess or leak, and to a have longer postoperative stay. Yamamoto et al,^8^ in a series of 343 patients who underwent 566 operations for primary or recurrent CD, reported that the preoperative presence of abscess or fistula at the time of laparotomy significantly increased the risk of septic complications after surgery. Alves et al^9^ found that presence of abscesses at the time of surgery significantly increased the risk of IAS complications after the first ileocecal resection. However, none of these studies compared the outcome of penetrating CD with or without IAS versus nonpenetrating lesions. The present study revealed that when penetrating CD is not accompanied with IAS, such as uncontrolled abscess or phlegmon, or free perforation, the postoperative outcome is similar to that for nonpenetrating CD.

The impact of PD of abscess on postoperative outcome in penetrating CD is still inconclusive. Poritz et al^10^ evaluated outcomes following treatment of Crohn IAS with a protocol consisting of PD, antibiotics, and PN, followed by surgery. Stoma creation was avoided in 84% of patients.^10^ Zerbib et al reported 78 patients with penetrating CD undergoing ileocecal resection after preoperative management consisting of bowel rest, nutritional therapy, intravenous antibiotics, weaning off steroids and immunosuppressants, and abscess drainage, low rates of postoperative morbidity, and fecal diversion were observed.^5^ In another study by Muller-Wille et al, PD of IAS before surgery significantly reduced the occurrence of severe postoperative IAS complications in patients with CD.^11^ On the contrary, in a series by Bafford et al, preoperative treatment of CD abscess with PD did not decrease the rate of postoperative complications and the rate of stoma creation.^6^ However, in Bafford's series, 31% of patients with technically successful PD had relapse of symptoms or abscess re-accumulation prior to surgery, whereas 26% of patients had clinical PD failure. The conflicting results among studies concerning PD may be explained by the fact that whether IAS was controlled by PD had a different impact on surgical outcome. Lobaton et al showed that surgery performed after PD failure resulted in a poorer outcome that for immediate surgery.^12^ The present study revealed that successful control of IAS significantly lowers the incidence of postoperative complications and stoma creation in penetrating CD.

Preoperative nutrition was given to a substantial percentage of patients prior to surgery in the present study. The reason for this is that malnutrition is common in CD patients. In addition, our strategy was to induce remission and wean off steroids and anti-TNF agents using EN in all patients preoperatively. Our previous studies have shown that disease activity affects postoperative recurrence and complications after bowel resection for CD.^13^ EN is less effective in inducing remission than corticosteroids,^14^ but unlike corticosteroids or anti-TNF agents, it did not increase postoperative infectious complications. We resected the diseased bowel even after IAS was controlled, as our previous data have shown that successful PD can provide safe anastomosis for resection, and a lower rate of stoma creation, but does not abolish the need for subsequent surgery.^15^ Lobaton et al revealed that PD provided durable abscess resolution in only one-third of patients.^12^

The lower rate of laparoscopic surgery among patients with penetrating CD was not surprising, especially patients with IAS. It is generally believed that laparoscopic surgery is associated with decreased perioperative complications,^16^ and the comparable postoperative outcome confirmed that penetrating CD without IAS is not a risk factor for postoperative complications.

A high rate of temporary stoma creation (30.6, 34.1, and 75.9%) in the current cohort is also remarkable. A stoma was constructed if a large residual abscess was present, the dissection was difficult, or if the nutritional status of the patient was poor. Previous studies have also reported that the stoma creation rate was high for patients underwent bowel resection for penetrating CD. In a prospective study by Goyer et al,^17^ 39% of patients undergoing laparoscopic ileocolonic resection with complex penetrating CD voluntarily performed a temporary diverting stoma. In another study by Melton et al, stoma creation was performed in 51% of patients with ileosigmoid fistula undergoing ileocolonic resection.^18^

The first limitation to this study included its retrospective design from a single center. Thus, the postoperative treatment was not controlled, which may have affected the outcomes. However, the strategy of preoperative management is standardized in our center including routine preoperative imaging evaluation, patients’ status optimization with nutritional support, appropriate antibiotics, use of percutaneous drains, and careful timing of surgery. Second, the rate of stoma creation was high for both penetrating and nonpenetrating CD, possibly because this is a referral center and a high percentage of patients were transferred in poor condition, and we chose not to perform primary anastomosis for those with a poor nutritional status and severe active disease.

## CONCLUSION

The results of present study revealed that penetrating CD with IAS, but not penetrating CD without IAS, is associated with high surgical risks in patients undergoing bowel resection. The result may revise the notion that all patients with penetrating CD have worse postoperative complications.
